# Role and mechanisms of vascular smooth muscle cell phenotypic transition in diabetic macrovascular complications

**DOI:** 10.1186/s40659-025-00665-8

**Published:** 2026-01-05

**Authors:** Qiyuan Yao, Hanyu Liu, Xueru Wang, Zehua Zhang, Hongyan Xie, Chunguang Xie, Hong Gao

**Affiliations:** 1 Hospital of Chengdu University of Traditional Chinese Medicine, Chengdu, 610072 Sichuan Province People’s Republic of China; 2 Chengdu University of Traditional Chinese Medicine, Chengdu, 610075 Sichuan Province People’s Republic of China; 3 TCM Prevention and Treatment of Metabolic and Chronic Diseases Key Laboratory of Sichuan Province, Hospital of Chengdu University of Traditional Chinese Medicine, Chengdu, 610072 Sichuan Province People’s Republic of China; 4 Department of Endocrinology, Hospital of Chengdu University of Traditional Chinese Medicine, Chengdu, 610072 Sichuan Province People’s Republic of China

**Keywords:** Vascular smooth muscle cells, Phenotypic transition, Diabetic macrovascular complications, Atherosclerosis

## Abstract

Diabetic macrovascular complications, as one of the leading causes of mortality in diabetic patients, are characterized by insidious onset and high residual risk. Effective prevention and treatment of diabetic macrovascular injury remain critical clinical challenges, necessitating the development of novel therapeutic strategies targeting this complication. Although recent studies have demonstrated that vascular remodeling driven by the phenotypic transition of vascular smooth muscle cells (VSMCs) plays a pivotal role in this process, and increasing attention has been paid to the metabolic pathways and mechanosensitive signaling pathways of VSMCs, the underlying molecular mechanisms remain incompletely elucidated. This review summarizes metabolic reprogramming in VSMCs in diabetes and its impact on vascular remodeling systematically, with a focus on elucidating the core mechanisms of endothelial-VSMC crosstalk, pathological characteristics of VSMCs phenotypic transition during different stages of atherosclerosis (AS), and potential diversified strategies such as the application of microRNAs, extracellular vesicle targeting techniques, and targeted protein regulation. Current evidence indicates that precise modulation of the metabolic-mechanical coupling network in VSMCs can significantly attenuate the progression of atherosclerotic plaques and enhance plaque stability. However, clinical translation remains challenged by insufficient targeting specificity and mechanistic complexity. Future studies should integrate multi-omics technologies with biomimetic models to further optimize therapeutic strategies.

## Introduction

Diabetes mellitus (DM) is an endocrine and metabolic disorder characterized by hyperglycemia. As one of the major global public health challenges, the number of adult diabetic patients aged ≥ 18 years worldwide surged from 200 million in 1990 to 828 million in 2022 approximately [1]. It is projected that 1.31 billion individuals (9.8% of the population) will be affected by this condition by 2050 [2]. DM could lead various complications, including macrovascular and microvascular diseases. Macrovascular complications [e.g., stroke, myocardial infarction (MI), and diabetic foot disease] are the primary causes of mortality and disability in diabetic patients, characterized by high mortality risk and early onset. The Framingham Study demonstrated that the risk of cardiovascular disease (CVD) in patients with type 2 diabetes mellitus (T2DM) is twice that of non-diabetic individuals [3]; Furthermore, 75% of diabetic patients die from cardiovascular complications [4, 5]. However, data from the UK Prospective Diabetes Study (UKPDS) indicate that intensive glucose-lowering therapy not only fails to effectively prevent macrovascular damage, but also induce hypoglycemia and increase the mortality rate of cardiovascular and cerebrovascular events [6–8]. Therefore, how to enhance macrovascular benefits in diabetic patients and effectively halt the progression of vascular damage at an early stage has become a major research focus and a critical clinical challenge that urgently requires breakthroughs.

VSMCs regulate blood flow distribution through contraction-relaxation cycles as the core component of the vascular tunica media, while secreting extracellular matrix (ECM) to maintain vascular elasticity [9]. These cells exhibit remarkable dynamic phenotypic plasticity, enabling dual roles in vascular homeostasis maintenance and injury repair [10–14]. In physiological conditions, the contractile phenotype of VSMCs precisely regulates vascular tension via calcium ion-dependent signaling networks (e.g., α-smooth muscle actin (α-SMA) mediated mechanical stress responses) [15]; Upon vascular injury, VSMCs undergo dedifferentiation, transitioning into a migratory-proliferative phenotype with upregulated expression/secretion of phosphatidylinositol 3-kinase gamma (PI3Kγ) [16] or matrix metalloproteinases (MMPs) [17, 18], which participate in intimal remodeling.

The phenotypic transition of VSMCs refers to the pathological transformation from a differentiated (contractile phenotype) to a dedifferentiated state (synthetic/migratory phenotype), characterized by downregulation of α-SMA expression and upregulated secretion of collagen/inflammatory factors [19]. As a pivotal event in vascular remodeling, this process facilitates injury repair through migration and proliferation during physiological vascular repair. However, with the chronic hyperglycemia, oxidative stress, advanced glycation end products (AGEs), and proinflammatory microenvironment unique to diabetes, VSMCs phenotypic transition is recognized as a key contributor to macrovascular complications in diabetes, particularly in both early- and late-stage AS [20, 21]. Although foam cells have traditionally been attributed to monocyte-derived macrophages, recent evidence underscores the important role of transdifferentiated VSMCs, which can adopt a macrophage-like phenotype and contribute to lipid accumulation within plaques. Lineage tracing studies in atherosclerotic mices have demonstrated that approximately 30–70% of the cellular population within atherosclerotic plaques originates from VSMCs [22–24]. In the pathophysiology of AS, VSMCs undergo a phenotypic transition from a contractile to a synthetic state, during which they secrete ECM components such as collagen and elastin. This process contributes to the stabilization of atherosclerotic plaques [25, 26]. Concurrently, under hypoxic and inflammatory conditions, VSMCs migrate from the tunica media to the intima, forming a fibrous cap that covers the lipid core and isolates inflammatory factors, thereby preventing plaque rupture [27, 28]. This mechanism exerts a protective effect during the early stages of AS. However, in advanced AS, VSMCs can undergo phenotypic switching into other cell types, such as macrophage-like cells [29–31], foam cells [32], mesenchymal stem cell-like cells [24], and osteochondrogenic cells [33]. The transformed VSMCs directly participate in pathological processes including atherosclerotic plaque formation, vascular calcification, and medial degeneration. Ultimately, these changes contribute to plaque destabilization and loss of vascular compliance, forming the cascading pathological basis of diabetic macrovascular complications from endothelial injury to vascular wall sclerosis [34]. Herein, this review systematically delineates the various phenotypes of VSMCs under physiological conditions and briefly discusses how multiple factors in the diabetic state collectively induce the phenotypic transition of VSMCs through complex feedback mechanisms. We cite authoritative review articles or books to provide broader context (Sect. 1 & 2). Furthermore, taking the phenotypic transition of VSMCs in diabetes as a starting point, we conducted a comprehensive review of existing research to connect the entire disease course of diabetic macrovascular complications—encompassing “endothelial injury–plaque formation–cardiovascular events/lower extremity arterial occlusion–failure of revascularization” (Sect. 3). Emphasis was placed on citing representative original studies and key reviews. Finally, a detailed summary of various therapeutic strategies and their corresponding clinical outcomes is provided (Sect. 4), aiming to offer a theoretical basis and potential breakthroughs for the current diagnosis and treatment paradigm of diabetic macrovascular complications. All cited references consist of specific research articles or clinical trial reports to enhance the empirical foundation and reference value of the content.

## Fundamental mechanisms of phenotypic transition in VSMCs

### Phenotypic and functional heterogeneity of VSMCs

**Fig. 1:**
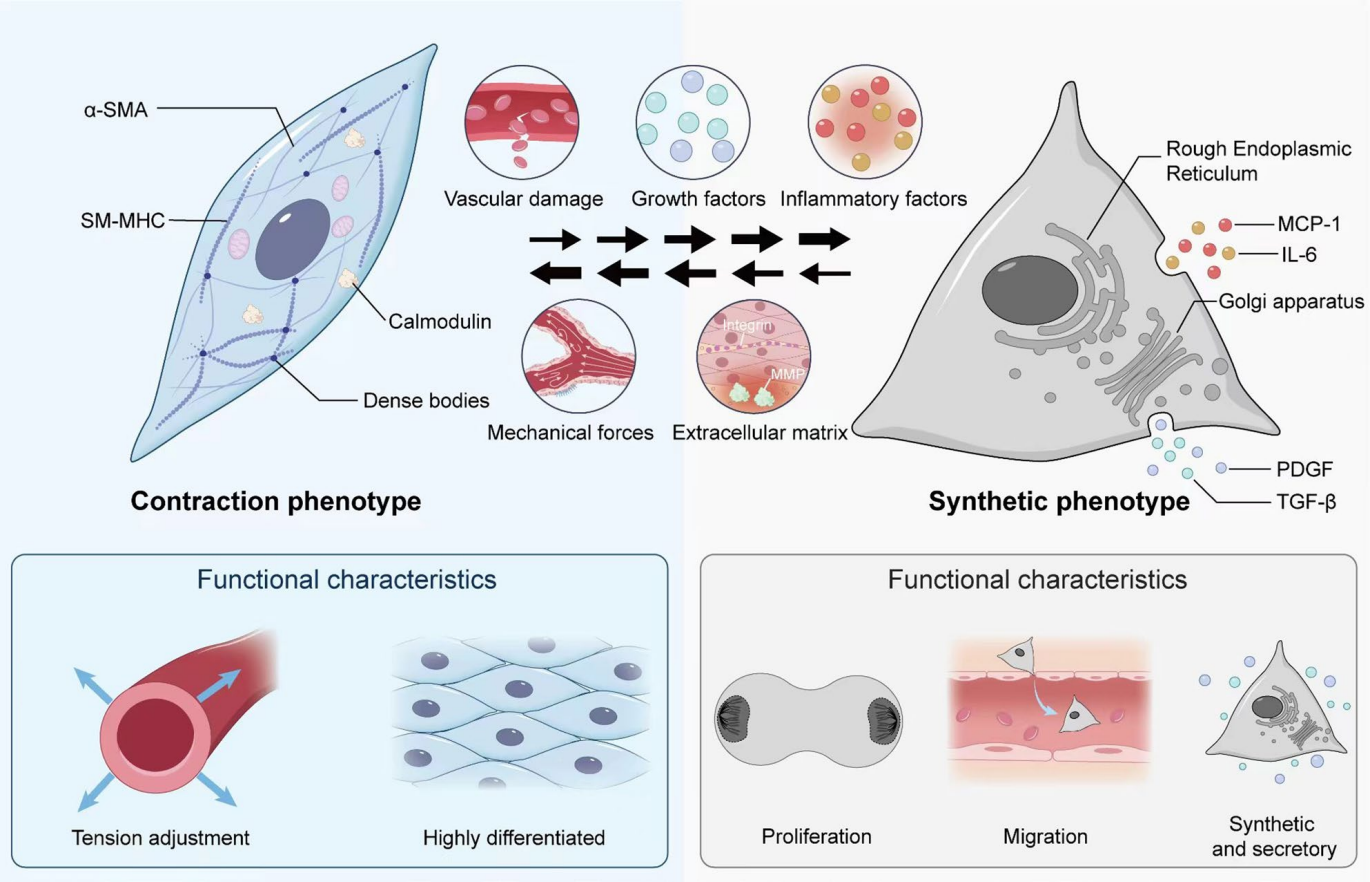
**Schematic Illustration of Contractile and Synthetic Phenotypes in VSMCs** VSMCs exhibit remarkable plasticity, with their phenotypic transition determined by complex interactions among multiple local environmental factors rather than any single determinant. Alterations in any of these factors may induce phenotypic transition in VSMCs. Contractile VSMCs express high levels of contractile-associated proteins, including α-SMA, SM-MHC, and calmodulin. Under physiological conditions, these molecules form a contractile myofilament network that enables VSMCs to precisely regulate vascular wall tension. In contrast, synthetic VSMCs show significant downregulation of contractile proteins but possess enhanced proliferative and migratory capabilities, playing a crucial role in vascular injury repair. These two phenotypes are interconvertible under certain conditions. Abbreviations: α-SMA: α-smooth muscle actin, SM-MHC: smooth muscle myosin heavy chain, MCP-1: monocyte chemoattractant protein-1, IL-6: interleukin-6, PDGF: platelet-derived growth factor, TGF-β: transforming growth factor-beta

Under normal physiological conditions, VSMCs exhibit a contractile phenotype predominantly [35]. This phenotype is characterized by high differentiation, intracellular enrichment of well-organized myofilaments, and elevated expression of contractile-associated proteins such as α-SMA, smooth muscle myosin heavy chain (SM-MHC), and calmodulin [36, 37]. These molecules collectively form an elaborate intracellular contractile myofilament network, enabling VSMCs to precisely regulate vascular wall tension through calcium ion-mediated signaling pathways (e.g., the myosin light chain kinase (MLCK) signaling pathway [38]), thereby maintaining normal vascular contractile function and hemodynamic stability. The functional core of the contractile phenotype in VSMCs lies in their mechano-chemical coupling response to vasomotor signals, ensuring precise matching between blood pressure and organ perfusion [39, 40]. In this state, VSMCs exhibit low proliferative and migratory activity while maintaining vascular wall structural integrity and elasticity through sustained cell-matrix connections (Fig. 1).

Under physiological repair or pathological conditions, a subset of VSMCs can undergo phenotypic switching to the synthetic phenotype [9]. These cells exhibit a less differentiated state characterized by significant downregulation of contractile proteins, yet demonstrate enhanced proliferative and migratory capacities [20, 21, 23, 41]. In synthetic VSMCs, the expansion of rough endoplasmic reticulum (ER) and Golgi apparatus facilitates the synthesis and secretion of ECM components, including collagen and elastin. Concurrently, these cells release various growth factors [platelet-derived growth factor (PDGF), transforming growth factor-beta (TGF-β)] and inflammatory mediators [interleukin-6 (IL-6), monocyte chemoattractant protein-1 (MCP-1)], thereby playing a pivotal role in vascular injury repair and adaptive remodeling [28, 42, 43]. Especially after acute injury, VSMCs adopt synthetic phenotype to facilitate tissue repair by migrating to the injured site, proliferating, and remodeling the extracellular environment [22, 23]. However, persistent or excessive activation of synthetic VSMCs may lead to abnormal ECM deposition and pathological vascular remodeling, which increases the risk of AS, aneurysms, and other pathological alterations [9, 44, 45].

In summary, the functional differences between the contractile and synthetic phenotypes of VSMCs are reflected in their distinct biological roles and regulatory mechanisms. VSMCs of the contractile phenotype primarily maintain vascular tone and stabilize blood pressure, whereas those of the synthetic phenotype play a pivotal role in vascular injury repair and inflammatory responses by participating in proliferation, migration, and ECM remodeling during vascular repairing and remodeling. The contractile phenotype of VSMCs exhibits greater functional stability and persistence, primarily maintaining basal vascular physiological activities. In contrast, the synthetic phenotype of VSMCs demonstrates higher responsiveness and dynamic characteristics, with its activity being regulated by injury signals and cytokines. The dynamic equilibrium between these two phenotypes is crucial for maintaining normal vascular physiological functions, structural homeostasis, and response to injury.

### **Phenotypic transition of VSMCs induced by DM**

Sustained hyperglycemia drives the phenotypic transition of VSMCs from contractile to synthetic phenotype through multiple molecular pathways, constituting central pathological mechanism of diabetic macrovascular complications. This process directly contributes to pathological vascular remodeling and accelerates the progression of AS by promoting proliferation, migration, and the release of inflammatory factors [46, 47]. Specifically, the mitochondrial electron transport chain generates excessive reactive oxygen species (ROS) under hyperglycemic conditions, while glucose metabolism through the polyol pathway is concurrently enhanced, leading to NADPH depletion and consequent reduction in antioxidant capacity. These ROS activate pro-proliferative signaling pathways such as mitogen-activated protein kinases (MAPK, including ERK1/2 and p38) and PI3K/Akt, thereby upregulating the expression of cell cycle proteins (e.g., Cyclin D1). This cascade ultimately drives VSMCs from the G1 phase into the S phase, resulting in VSMCs proliferation [48]. In addition, hyperglycemia promotes the formation of AGEs through non-enzymatic reactions (Maillard reaction). The binding of AGEs to their receptor (RAGE) induces receptor oligomerization [49], thereby activating multiple signaling pathways, including the NADPH oxidase (NOX4) [50] and protein kinase C (PKC) pathways [51, 52]. Concurrently, the nuclear translocation of nuclear factor erythroid 2-related factor 2 (Nrf2) is inhibited, leading to diminished expression of antioxidant enzymes such as superoxide dismutase (SOD) and glutathione peroxidase (GPx). Consequently, the redox homeostasis is disrupted, further exacerbating oxidative stress [53]. This process promotes the proliferation, migration, and enhanced synthetic function of VSMCs. Moreover, AGEs can directly interact with the vascular wall matrix, altering its elasticity and stability, thereby aggravating vascular stiffening and stenosis [54].

Under hyperglycemic conditions, the role of insulin undergoes significant alterations. Although insulin maintains normal VSMCs function via the PI3K/Akt signaling pathway under physiological conditions, excessive or aberrant insulin responses during hyperglycemia with concomitant insulin resistance may promote VSMCs dedifferentiation and proliferation [55, 56] through overactivation of signaling pathways such as mTOR and ERK1/2 [57]. This process not only accelerates vascular remodeling but may also exacerbate endothelial injury and inflammatory responses through insulin-mediated upregulation of vascular endothelial growth factor (VEGF) and MMPs [58].

In addition, inflammatory cytokines play a profound role in high glucose-induced phenotypic transition of VSMCs. Under hyperglycemic conditions, chronic low-grade inflammation in the body activates immune cells such as macrophages and T cells, leading to the release of abundant inflammatory cytokines, including tumor necrosis factor-alpha (TNF-α), IL-6, and interleukin-1 beta (IL-1β) [59]. These cytokines not only promote endothelial cells (ECs) injury and leukocyte adhesion but also stimulate receptors on VSMCs (e.g., TNF-R1, IL-1R) to activate signaling pathways such as nuclear factor-kappa B (NF-κB) and MAPK, thereby inducing the shift of VSMCs toward the synthetic phenotype [60]. Furthermore, the accumulation of inflammatory cytokines enhances the uptake of low-density lipoprotein (LDL) by VSMCs, contributing to foam cell formation and exacerbating AS plaque development and vascular wall fibrosis [19, 24].

These factors interact through intricate feedback mechanisms, driving the phenotypic transition of VSMCs from contractile to synthetic state, thereby establishing the diabetes-specific vascular pathological profile. This phenotypic shift not only exerts profound effects on vascular wall structure and function but also plays a pivotal role in the initiation and progression of AS and pathological vascular remodeling.

## The role of VSMCs phenotypic transition in diabetic macrovascular complications

Phenotypic transition of VSMCs serves as a pivotal regulatory hub in the malignant progression of diabetic macrovascular complications [20, 21, 41]. During the endothelial injury phase, hyperglycemia and the AGEs-RAGE axis induce an imbalance in endothelial-derived factors [e.g., platelet-derived growth factor (PDGF-BB)] via oxidative stress, triggering the shift of VSMCs toward synthetic phenotype. This transition is characterized by the loss of contractile properties and the acquisition of enhanced proliferative and migratory capacities [61]. In the progression of AS, synthetic VSMCs infiltrate the intima, secrete pro-inflammatory mediators, and engulf lipids to form foam-like cells, thereby promoting plaque instability and fibrous cap rupture. These mechanisms directly exacerbate the risk of coronary plaque rupture in diabetic patients [21, 24]. In the context of persistent hyperglycemia and a residual inflammatory microenvironment following percutaneous intervention, synthetic VSMCs drive excessive neointimal hyperplasia and matrix calcification, ultimately leading to restenosis and vascular compensatory failure [62]. This dynamic phenotypic plasticity not only connects the entire disease progression from “endothelial injury-plaque formation-cardiovascular events/lower extremity arterial occlusion-revascularization failure”, but also through multi-axis interactions of metabolic-inflammatory-mechanical signals, renders diabetic macrovascular complications an “irreversible” pathological loop.

### Vascular endothelial injury and phenotypic transition of VSMCs

Vascular endothelial injury and phenotypic transition of VSMCs form a synergistic vicious cycle in vascular pathological progression. Following endothelial injury, the loss of barrier function disrupts vascular homeostasis, leading to the release of various bioactive mediators (e.g. PDGF, TGF-β, etc.) [63, 64]. These molecules drive the phenotypic transition of VSMCs from contractile to synthetic phenotype via direct intercellular communication or paracrine signaling pathways. Synthetic phenotype VSMCs exacerbate endothelial barrier disruption and promote vascular wall stiffening and plaque formation through the secretion of inflammatory mediators and aberrant ECM remodeling, thereby accelerating the progression of AS, coronary artery disease (CAD), and post-interventional restenosis [19, 28, 65, 66]. The dynamic interplay between these processes constitutes a central pathogenic axis in vascular disorders, disrupting the structural and functional homeostasis of blood vessels. This interaction represents a pivotal mechanism underlying the pathological progression of AS and vascular remodeling.

#### The initiating role of endothelial dysfunction in diabetic macrovascular complications

**Fig. 2:**
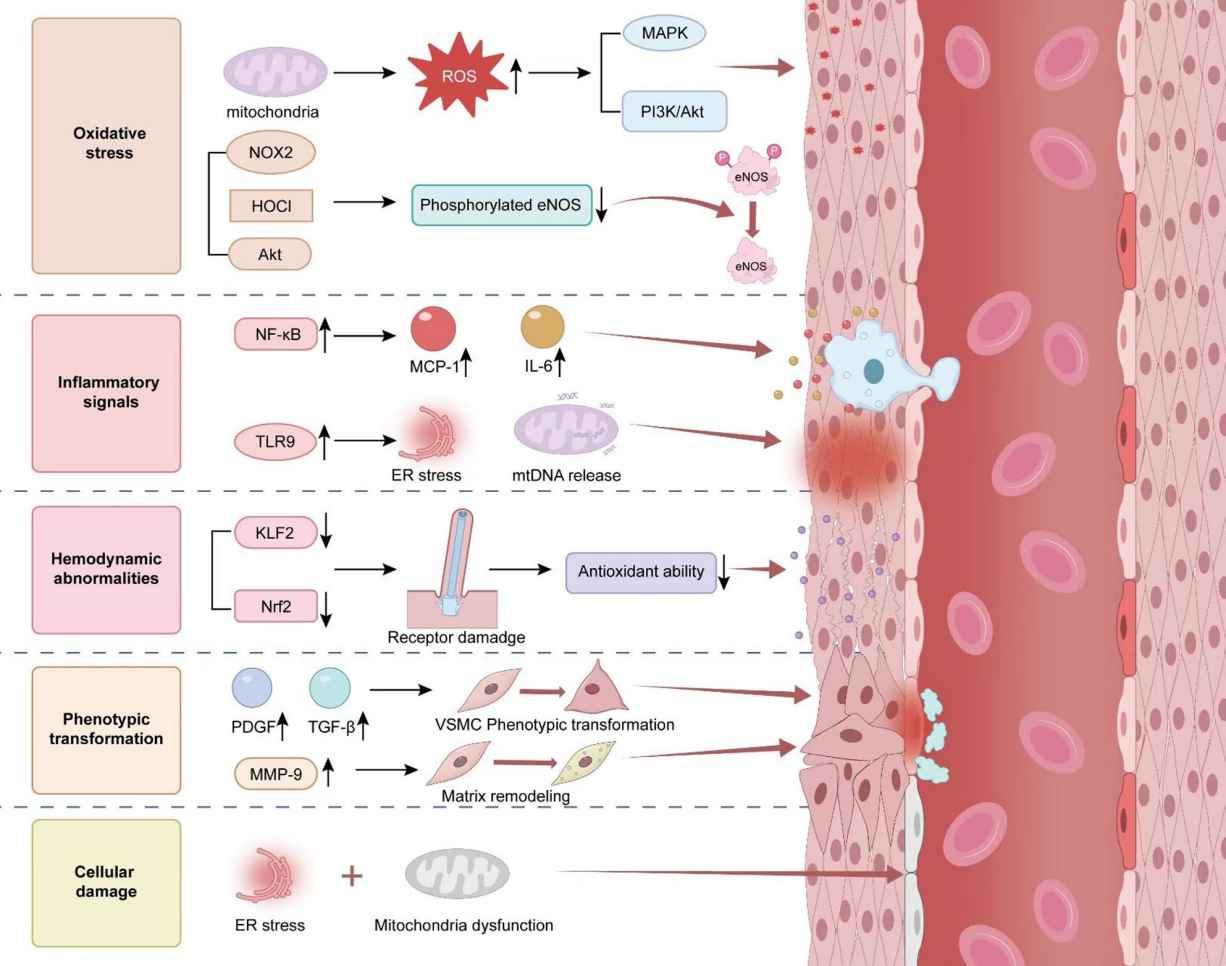
**Schematic Illustration of the Mechanisms Underlying Vascular Endothelial Injury and VSMCs Phenotypic Transition** Hyperglycemia induces mitochondrial oxidative stress through metabolic dysregulation pathways such as the polyol pathway and hexosamine pathway, leading to ROS generation and activation of the NF-κB inflammatory signaling cascade. This process promotes ECs to secrete MCP-1 and IL-6, which recruit monocytes/macrophages to infiltrate the vascular wall. ER stress and mitochondrial dysfunction trigger endothelial apoptosis, releasing cell-derived microparticles and mtDNA that activate TLR9 signaling, thereby amplifying local inflammation. Furthermore, hemodynamic disturbances (e.g., aberrant shear stress) impair the mechanosensory function of endothelial cilia, downregulating the activity of KLF2 and Nrf2, thereby weakening antioxidant defense mechanisms. These alterations stimulate ECs to release PDGF and TGF-β, inducing VSMCs to undergo a phenotypic transition toward a synthetic phenotype. Subsequently, VSMCs secrete MMP-9, facilitating ECM remodeling. Abbreviations: ROS: reactive oxygen species, MAPK: mitogen-activated protein kinases, PI3K/Akt: phosphatidylinositol 3-kinase/protein kinase B, eNOS: endothelial nitric oxide synthase, NF-κB: nuclear factor-kappa B, MCP-1: monocyte chemoattractant protein-1, IL-6: interleukin-6, ER: endoplasmic reticulum, mtDNA: mitochondrial DNA, KLF2: Krüppel-like factor 2, Nrf2: nuclear factor erythroid 2-related factor 2, PDGF: platelet-derived growth factor, TGF-β: transforming growth factor-beta, MMP-9: matrix metalloproteinase-9

Endothelial dysfunction represents both the initial trigger and a central pathogenic mechanism in the development of diabetic macrovascular complications [67]. Hyperglycemia triggers mitochondrial oxidative stress by disrupting metabolic pathways, including the polyol and hexosamine pathways [47, 68], which promotes ROS production and activates the NF-κB inflammatory signaling cascade. This process stimulates ECs to secrete MCP-1 [69, 70] and IL-6 [71], thereby recruiting monocytes/macrophages to infiltrate the vascular wall [72]. Simultaneously, peroxidasin [(PXDN), e.g., VPO1] attenuates the phosphorylation of endothelial nitric oxide synthase (eNOS) at the Ser1177 site through the NOX2/HOCl/Akt pathway, exacerbating AGEs-induced endothelial dysfunction in diabetic vasculature [73]. Hemodynamic disturbances such as abnormal shear stress downregulate the activity of Krüppel-like factor 2 (KLF2) [74] and Nrf2 [75] by impairing endothelial cilia mechanoreceptors, that weaken antioxidant defenses. These alterations promote the phenotypic transition of ECs toward a procoagulant and proinflammatory state, accompanied by the release of PDGF and TGF-β, which stimulate synthetic transformation of VSMCs. Concurrently, matrix metalloproteinase-9 (MMP-9) secretion is enhanced, which facilitates ECM remodeling [18, 76, 77]. In addition, ER stress and mitochondrial dysfunction trigger endothelial apoptosis [78], activating Toll-like receptor 9 (TLR9) [79] signaling through the release of cellular microparticles and mitochondrial DNA (mtDNA) [80], which intensifies local inflammation. These responses not only cause direct vascular wall damage, but also exacerbate inflammation and oxidative stress through “endothelial-smooth muscle-immune cell” network, which establish the pathological foundation of diabetic macrovascular complications (Figs. 2 and 3).

#### Core mechanisms of Endothelial-VSMC interactions

**Fig. 3:**
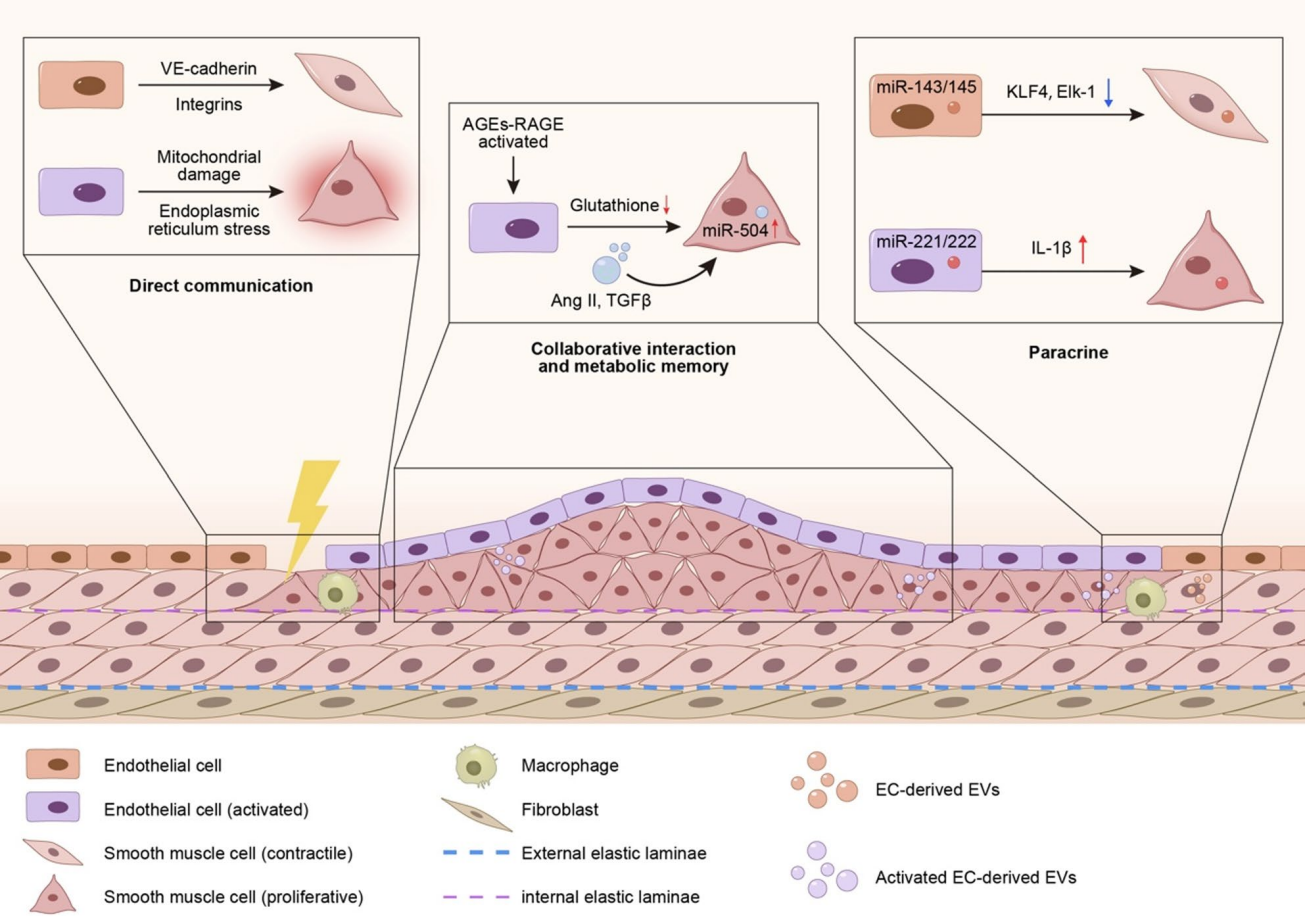
**Schematic Illustration of Core Mechanisms Underlying ECs-VSMCs Interactions** This figure illustrates three potential mechanisms of ECs-VSMCs crosstalk: (1) Direct Communication: Under physiological conditions, ECs and VSMCs maintain tight spatial coupling through direct intercellular contact and mechanical force transmission, ensuring vascular tone homeostasis. However, in diabetic patients, hyperglycemia induces mitochondrial dysfunction, leading to insufficient ATP synthesis and impairing EC responsiveness to hemodynamic shear stress. Concurrently, glucotoxicity-triggered ER stress downregulates cadherin expression, compromising gap junction functionality and directly contributing to dysregulated vascular tone. (2) Paracrine Signaling: Under physiological conditions, EC-derived exosomes are enriched with miR-143/145, which maintain the contractile phenotype of VSMCs by targeting and suppressing KLF4 and Elk-1. In contrast, hyperglycemia-induced exosomes exhibit elevated levels of miR-221/222. These exosomes not only promote EC activation but also enhance the inflammatory polarization of macrophages, thereby accelerating atherogenesis. (3) Synergistic Crosstalk and Metabolic Memory: The AGEs-RAGE signaling axis activates ECs to secrete Ang II and TGF-β, inducing a synthetic phenotypic switch in VSMCs and stimulating excessive collagen synthesis. Meanwhile, sustained upregulation of miR-504 suppresses the expression of VSMC contractile genes, driving their transition toward a pro-atherogenic phenotype. Additionally, heterogeneous populations of EVs secreted by various vascular wall cells appear to play a pivotal role in the progression of neointimal thickening. Abbreviations: VE: vascular endothelial, AGEs-RAGE: advanced glycation end products-receptor of advanced glycation end products, Ang II: angiotensin II, TGF-β: transforming growth factor-beta, KLF4: Krüppel-like factor 4, Elk-1: ETS domain-containing protein-1, IL-1β: interleukin-1 beta, EVs: extracellular vesicles

##### Dysregulation of direct intercellular communication and mechanical coupling

ECs and VSMCs establish tight spatial coordination through direct physical interactions [81] and mechanical force transmission [82]. Cadherin-based adherens junctions, particularly VE-cadherin, along with integrins, provide structural stability while facilitating mechanotransduction of hemodynamic shear stress and vascular wall tension through mechanosensitive channels like Piezo1 [83, 84] and subsequent cytoskeletal remodeling [85]. Under physiological conditions, mechanical coupling maintains vascular tone homeostasis [86]. However, hyperglycemia disrupts this equilibrium by triggering mitochondrial oxidative stress pathways [87]. Mitochondrial impairment reduces ATP production, diminishing endothelial cell responsiveness to shear stress. Glucotoxicity simultaneously induces ER stress, suppressing cadherin proteins such as N-cadherin and impairing gap junction function mediated by Connexin 43. These pathological changes ultimately dysregulate vascular tone control [88]. In the inflammatory microenvironment, infiltrated M1-type macrophages release pro-inflammatory cytokines such as TNF-α and IL-1β [89], which further suppress the bioavailability of endothelial nitric oxide (NO) while promoting excessive activation of the Rho kinase pathway in VSMCs, which lead to abnormal vasoconstriction and increased vascular stiffness [90]. The synergistic effect of disrupted mechanical signal transmission and immune-inflammatory responses exacerbates mechanical stress imbalance and fibrotic remodeling in the vascular wall.

##### Paracrine Signaling-Mediated remote regulation and inflammatory cascade

ECs and VSMCs form a dynamic interactive network through paracrine signaling, which drives pathological vascular remodeling in diabetic macrovascular complications [91]. Endothelin-1 (ET-1), as a core paracrine mediator [92], initiates the ERK1/2 signaling pathway through ETA receptors, promoting Cyclin D1-dependent proliferation [93] and RhoA/ROCK-mediated migration of VSMCs [94]. Exosomes play a dynamic regulatory role in this process: under physiological conditions, endothelial-derived exosomes containing miR-143/145 maintain the contractile phenotype of VSMCs by targeting KLF4 and Elk-1 [95]; whereas under hyperglycemic conditions, induced exosomes are enriched with miR-221/222, and these exosomes promote AS formation by enhancing endothelial activation and inflammatory polarization of macrophages [96]. Furthermore, endothelial cell-derived extracellular vesicles (EVs) promote VSMCs calcification under high-glucose conditions through the circ_0008362/miR-1251-5p/Runx2 axis. In addition, patients with elevated plasma EV levels were significantly associated with more severe coronary artery and aortic calcification [97]. It is noteworthy that the metabolic-inflammatory cascade triggered by mitochondrial dysfunction further amplifies pathological progression. Hyperglycemia induces mitochondrial fragmentation in ECs [98, 99], leading to the release of oxidatively damaged mtDNA, which is subsequently transported to VSMCs [100]. Concurrently, oxidatively modified proteins such as TXNIP, delivered via exosomal surface integrin αvβ3 [101] promote substantial IL-1β secretion through NOD-like receptor protein 3 (NLRP3) inflammasome activation [102]. These processes collectively establish a synergistic network characterized by “proliferation-metabolic dysregulation-inflammatory amplification,” ultimately fostering a profibrotic microenvironment that transforms localized endothelial injury into systemic vascular remodeling.

##### Synergistic interaction between direct communication and paracrine signaling

In the endothelial-VSMC interaction network, direct communication and paracrine signaling do not operate in isolation but collaboratively regulate vascular function through spatiotemporal coordination. On the one hand, hyperglycemia disrupts the transfer of protective metabolites such as glutathione from ECs to VSMCs by shortening the half-life of gap junctions, while simultaneously facilitating the diffusion of pro-apoptotic factors including caspase-3 [103]. On the other hand, the AGEs-RAGE axis-activated ECs secrete angiotensin II (Ang II) and TGF-β, which induce the synthetic phenotype transition of VSMCs and enhance collagen synthesis in a paracrine manner [104]. Furthermore, diabetes-associated chronic inflammation establishes a “metabolic-inflammatory memory“ [105] through epigenetic modifications and non-coding RNA-mediated mechanisms. Even after glycemic control is achieved, the aberrant endothelial-VSMC crosstalk persists, leading to irreversible vascular remodeling. Reddy MA and colleagues performed small RNA sequencing (smRNA-seq) analysis by comparing VSMCs from db/db mice with those from db/+ mice, identifying multiple differentially expressed miRNAs in VSMCs under diabetic conditions. Further functional characterization was conducted on miR-504, which was found to be upregulated in db/db VSMCs. This study [106] revealed that miR-504 was persistently upregulated in VSMCs of diabetic db/db mice and maintained high expression levels even after multiple passages of ex vivo culture. Growth factor receptor-bound protein 10 (Grb10) was identified as a critical downstream mediator of miR-504 function. Mechanistically, miR-504 enhanced PDGF-induced ERK1/2 activation by downregulating Grb10, concurrently suppressing the expression of VSMC-specific contractile genes while elevating proinflammatory markers. This regulatory cascade ultimately promoted the phenotypic transition of VSMCs toward a pro-AS state. The resulting dysregulation promoted pathological neovascularization in atherosclerotic lesions and chronic vascular fibrotic remodeling.

### AS and phenotypic transition of VSMCs

**Fig. 4:**
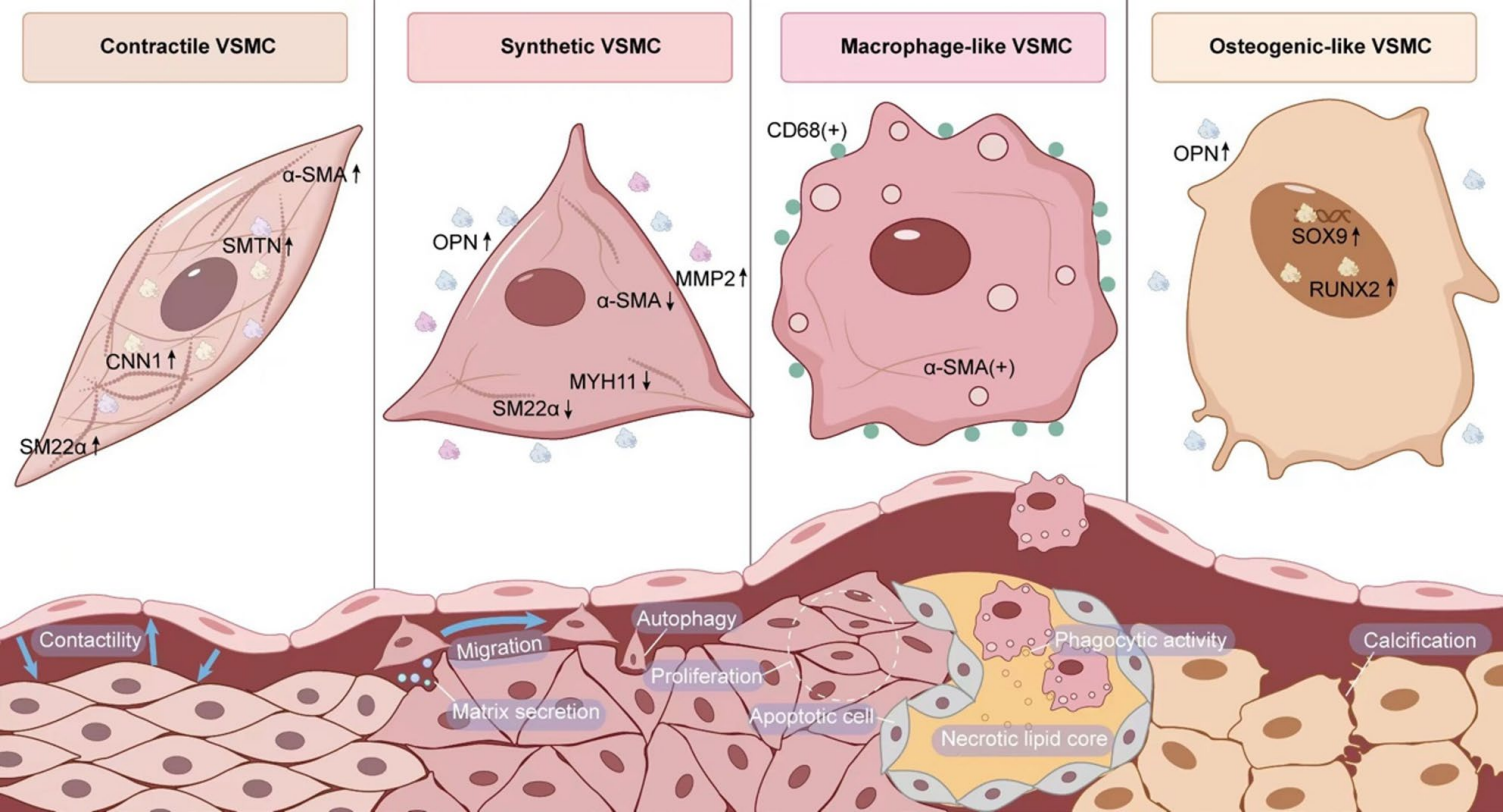
**Schematic Illustration of Phenotypic Characteristics of VSMCs at Different Stages of AS** The medial layer of normal blood vessels is composed of contractile phenotype VSMCs, which exhibit strong contractile capacity and high expression of contractile phenotype markers. Following endothelial injury, lipoproteins begin to accumulate in the subendothelial space, and ROS generated during AS promote the formation of ox-LDL. The damaged endothelial cells also highly express adhesion molecules that recruit circulating monocytes, facilitating their infiltration into the vascular wall and subsequent differentiation into macrophages. These macrophages engulf large amounts of lipids, transforming into foam cells, which contribute to the formation of the necrotic lipid core within AS plaques. On the other hand, VSMCs in the medial layer undergo dedifferentiation and migrate into the intima, where they form myogenic foam cells through lineage transdifferentiation. These cells acquire the characteristic foam cell morphology via lipid uptake mechanisms similar to those observed in macrophages. Concurrently, membrane-bound microvesicles released during apoptosis serve as templates for crystal deposition and calcification, accelerating the formation of microcalcifications within plaques. Microcalcifications induce localized mechanical stress concentration, promoting fibrous cap rupture. Osteoblast-like phenotype VSMCs exhibit pro-calcific properties and actively participate in plaque calcification, thereby accelerating disease progression. Abbreviations: α-SMA: α-smooth muscle actin, SMTN: smoothelin, CNN1: Calponin 1, SM22α: smooth muscle 22 alpha, OPN: osteopontin, MYH11: myosin heavy chain 11, MMP2: matrix metalloproteinase 2, SOX9: sex-determining region Y box protein 9, RUNX2: runt-related transcription factor 2

As the core pathological basis of diabetic macrovascular complications, AS is fundamentally characterized by an aberrant vascular repair response to chronic injury. This process involves multilevel pathophysiological remodeling, including oxidative stress-induced endothelial barrier disruption [107], inflammation [108], and dysregulation of the immune microenvironment [109]. Recent studies have demonstrated that VSMCs actively contribute to AS progression rather than serving as “passive participants”—they dynamically regulate disease progression through phenotypic switching [23, 110–112]. With pathological stimuli such as oxidized low-density lipoprotein (ox-LDL) [113], inflammatory cytokines (TNF-α, IL-1β), and hemodynamic disturbances, VSMCs undergo lineage reprogramming, transitioning into synthetic, macrophage-like, or osteoblast-like phenotypes [24, 28, 114, 115]. This phenotypic transition not only drives cell migration into the intima and promotes aberrant proliferation, leading to fibrous cap thickening and ECM remodeling, but also contributes to lipid-rich necrotic core formation through cholesterol uptake, generating myogenic foam cells. These cells, together with monocyte-derived foam cells, constitute heterogeneous sources of necrotic lipids [116]. It should be noted that foam cells represent a functional phenotype that can be acquired either by monocytes (differentiating into macrophages) or through phenotypic transition of VSMCs. The distinction lies in the fact that monocyte-derived foam cells primarily drive early inflammatory progression, whereas in advanced stages, smooth muscle-derived foam cells may exert more significant effects on fibrosis and plaque stability. Lineage tracing studies have demonstrated that VSMC-derived foam cells can account for 50%–70% of all foam cells in advanced plaques [29, 32]. It is noteworthy that phenotypic transition exerts a dual-edged effect: it may initially stabilize the fibrous cap and delay plaque rupture in the early stages [117], whereas sustained activation ultimately promotes calcified nodule formation and increases plaque vulnerability [118]. Emerging evidence from spatial transcriptomics further reveals that unstable AS plaques express a distinct pro-inflammatory transcriptional signature, with regional upregulation of pro-inflammatory mediators, such as interferon-gamma (IFN-γ), major histocompatibility complex (MHC) class II molecules, and pro-inflammatory cytokines, and differential activation of pro-thrombotic signaling pathways [119]. Therefore, elucidating the molecular switches that govern phenotypic transitions in VSMCs and their epigenetic regulatory network not only provides novel insights into the pathophysiology of AS, but also establishes a theoretical foundation for developing precision intervention strategies targeting cellular plasticity (Fig. 4).

#### Plaque formation stage: synthetic phenotype-driven intimal hyperplasia and lipid deposition

During the early stages of atherosclerotic plaque formation, the phenotypic transition of VSMCs toward a synthetic phenotype serves as a pivotal mechanism, which critically contributes to plaque initiation and progression by promoting migration, proliferation, and ECM remodeling [120]. Following endothelial injury, locally released PDGF-BB downregulates contractile markers (α-SMA, SM22α) while upregulating the expression of migration-related molecules such as RhoA/ROCK and MMPs, along with cell cycle regulators like Cyclin D1. This promotes the migration of VSMCs from the medial layer to the intima and drives their aberrant proliferation, leading to pathological intimal thickening. Concurrently, VSMCs secrete abundant ECM, which form the fibrous scaffold of atherosclerotic plaques [121–123]. In the diabetic microenvironment, elevated ox-LDL synergizes with glycated ECM components to activate Toll-like receptors (TLRs), thereby further promoting the secretion of chemokines (e.g., MCP-1) by VSMCs. This cascade recruits monocytes and facilitates their differentiation into foam cells, ultimately accelerating the formation of lipid cores [124, 125]. Notably, diabetes-specific metabolic disorders such as insulin resistance exacerbate plaque vulnerability by upregulating the CX3CL1/CX3CR1 axis, which is closely associated with reduced VSMCs survival [126].

#### Plaque stabilization phase: phenotypic imbalance and fibrous cap structural disruption

During the plaque stabilization phase, VSMCs maintain the mechanical integrity of the fibrous cap by secreting collagen and elastic fibers [115]. However, persistent hyperglycemia in diabetes triggers mitochondrial dysfunction, leading to insufficient ATP synthesis and a burst of mitochondrial reactive oxygen species (mtROS), which accelerates VSMCs senescence and suppresses autophagy [111, 127]. This metabolic stress shifts VSMCs toward a synthetic phenotype—resulting in excessive secretion of matrix metalloproteinases (e.g., MMP-2/9) that degrade the ECM, coupled with a decline in collagen synthesis capacity. More importantly, diabetes-associated chronic inflammation induces the expression of pro-inflammatory factors (e.g., IL-1β, TNF-α) in VSMCs [128], establishing a positive feedback loop of “inflammation-ECM degradation” [129]. Furthermore, AGE-RAGE binding induces ER dysfunction in VSMCs, which diminishes cellular density within the fibrous cap and lowers the critical threshold for plaque stability [130]. The resulting structural defects render the plaque vulnerable to rupture under hemodynamic stress.

#### Plaque rupture phase: terminal events mediated by apoptosis and calcification

During the plaque rupture phase, apoptosis and calcification within atherosclerotic plaques are recognized as key drivers of terminal events, such as acute coronary syndrome and MI. As the disease advances, a significant number of foam cells (including macrophages and smooth muscle cells) in the plaque core undergo apoptosis. If the phagocytic clearance mechanism such as effector phagocytosis is impaired, apoptotic remnants accumulate, which result in necrotic core expansion and further destabilization of the plaque [131–134]. Meanwhile, the membranous vesicles released during apoptosis serve as templates for crystal deposition and calcification, accelerating the formation of microcalcifications within plaques [135]. These microcalcifications, predominantly distributed beneath the fibrous cap, induce localized mechanical stress concentration in this region, promoting fibrous cap rupture [136, 137]. When the plaque ruptures, the exposure of plaque contents to blood flow triggers platelet aggregation and procoagulant responses, ultimately leading to acute thrombosis formation [138, 139]. It is noteworthy that osteoblast-like phenotypic VSMCs exhibit pro-calcific properties by expressing osteogenic-related genes such as SOX9 and Runt-related transcription factor 2 (Runx2), which actively participate in plaque calcification. Consequently, the phenotypic transition of VSMCs synergizes with their apoptosis to accelerate fibrous cap thinning and calcification, representing a critical pathological mechanism underlying the onset of ischemic terminal events.

### Restenosis after interventional procedures and phenotypic transition of VSMCs

Patients with diabetes and concurrent AS face a 2–3 times higher risk of restenosis following vascular intervention compared to non-diabetic populations [140–142]. The underlying mechanism primarily involves aberrant phenotypic transition of VSMCs, driven by the combined effects of hyperglycemia, AGEs, insulin resistance, and platelet dysfunction [143–146]. Although the application of drug-coated balloons (DCBs) or drug-eluting stents (DESs) has been demonstrated to potentially improve post-endovascular treatment patency [147–150], the incidence of restenosis remains considerably high. Clinical epidemiological data indicate that the in-stent restenosis (ISR) rate in diabetic patients subjected to percutaneous coronary intervention (PCI) reaches 10–20% [151, 152]. Notably, poorly controlled diabetic patients (HbA1c > 7%) exhibit a 2.54-fold higher risk of ISR compared with those maintaining HbA1c ≤ 7% [153]. This phenomenon is closely associated with the metabolic disorders characteristic of DM: chronic hyperglycemia induces excessive accumulation of ROS through mitochondrial electron leakage and NADPH oxidase activation, subsequently triggering oxidative DNA damage and aberrant epigenetic modifications [154–156]. Notably, clinical evidence have demonstrated that hyperglycemia remains significantly correlated with restenosis occurrence even in non-DM individuals [157]. Further studies untangled the pivotal role of AGEs in this process. By forming cross-links with ECM proteins such as collagen and laminin in the vascular wall, AGEs elevate vascular stiffness and mechanical stress, which may exacerbate vascular remodeling and stenosis following interventional therapy.A study by Liu et al. [158] revealed that high mobility group box 2 (HMGB2) promotes the generation of ROS via the receptor for RAGE signaling in ischemic myocardium, thereby exacerbating cellular apoptosis, inflammation, and impaired autophagic clearance. This process involves p47phox-mediated elevation of ROS production, subsequently activating signaling pathways such as ERK, p38, and Akt, thereby upregulating the expression of genes associated with VSMC proliferation (e.g., cyclin D1) and migration (e.g., MMP2 and MMP9) [159]. Meanwhile, insulin resistance independently predicts early postoperative restenosis after coronary stent implantation by triggering metabolic reprogramming that suppresses the PI3K/Akt pathway through IRS-1 serine phosphorylation [160]. This process concurrently attenuates FoxO1-mediated antioxidant defense mechanisms while activating MAPK to promote VSMCs into an anabolic state [161, 162]. The prospective AIRE study revealed a strong association between insulin resistance and elevated post-interventional restenosis risk in T2DM patients, primarily mediated through aggravated vascular inflammation and intimal hyperplasia [163]. Furthermore, diabetes-specific platelet dysfunction aggravates the phenotypic imbalance of VSMCs through impaired mitochondrial energy metabolism. Diminished platelet mitochondrial complex III activity leads to significantly decreased ATP production efficiency, accompanied by increased ROS release, which promotes elevated secretion of N-formyl-l-methionine and methionine, ultimately driving VSMCs toward a synthetic phenotype [164–166].

## Potential therapeutic strategies targeting VSMCs phenotypic transition in diabetic macrovascular complications

Diabetic macrovascular complications are characterized by accelerated progression of AS, predominantly affecting the cardiovascular, cerebrovascular, and peripheral vascular systems. The pathological basis involves systemic dysregulation of vascular homeostasis, in which aberrant phenotypic plasticity of VSMCs plays a pivotal role. Consequently, multidimensional intervention strategies targeting VSMCs plasticity are emerging as novel approaches to overcome the limitations of current therapies. Herein, we summarize several pharmacological agents capable of modulating VSMCs phenotypic transition and potentially mitigating diabetic macrovascular complications. Furthermore, we also discuss the molecular mechanisms underlying the targeted regulation of VSMCs fate-determining factors, providing a theoretical foundation and innovative insights for improving vascular outcomes in diabetes.

### Emerging/classic anti-hyperglycemic medications

Hyperglycemia induces VSMCs dedifferentiation through oxidative stress, AGEs accumulation, and inflammatory activation, leading to aberrant proliferation, migration, and ECM secretion. Anti-hyperglycemic medications not only mitigate such damage by improving glycemic control but may also directly influence molecular mechanisms governing VSMCs phenotypic switching.

#### Glucagon-like peptide 1 receptor agonists(GLP1RAs)

GLP1RAs exert dual therapeutic effects on glycemic control and vascular protection through multi-target mechanisms. Their glucose-lowering efficacy primarily stems from precise regulation of pancreatic islet function: activation of β-cells enhances glucose-dependent insulin secretion, while suppression of α-cell glucagon release reduces hepatic glucose output; Concurrently, delayed gastric emptying and central actions on hypothalamic feeding centers contribute to improved postprandial glycemic variability [167]. These mechanisms have been consistently validated in multiple large-scale clinical trials, including the LEADER trial, SUSTAIN-6 study, and AWARD program [168–170].

More importantly, GLP1RAs suppress the pathological phenotypic transition of VSMCs through both direct and indirect pathways: On the one hand, they attenuate oxidative stress via endothelium-dependent mechanisms such as improving vasodilation by activating the AMPK/Akt/eNOS pathway, which indirectly suppresses VSMCs inflammatory activation [171]. On the other hand, they directly bind to glucagon-like peptide 1 (GLP-1) receptors on VSMCs, suppressing both ERK1/2 and PI3K/Akt signaling pathways, which mitigates high glucose-induced aberrant migration, proliferation, and apoptosis of VSMCs [172]. Furthermore, GLP1RAs suppress NADPH oxidase NOX4 activity via the AMPK-dependent pathway, reducing ROS generation while concurrently downregulating the expression of IL-6, MCP-1, and MMP-2/9 [173, 174]. These effects collectively inhibit VSMCs migration and neointimal hyperplasia. Additional evidence demonstrates that GLP1RAs such as liraglutide downregulate prolyl 4-hydroxylase subunit alpha-1 (P4HA1) expression through inhibition of the CD36-JNK-AP1 pathway, consequently attenuating myocardial fibrosis [175]. This multidimensional action—spanning from metabolism regulation to vascular homeostasis maintenance—highlights the unique therapeutic value of GLP1RAs in diabetic macrovascular complications (Tables 1 and 2).

**Table 1:** Clinical studies of GLP1RAs for the treatment of DM or DM-related diseases

Generic name	First author	Year	Disease	Model	Findings	Refs.
Dulaglutide	Gilles R Dagenais	2020	T2DM	Human	Dulaglutide demonstrated significant reduction in total cardiovascular events or fatal outcomes among T2DM populations with moderate cardiovascular risk	[176]
	Antonino Tuttolomondo	2021	T2DM	Human	Dulaglutide exerted favorable effects on arterial stiffness and endothelial function parameters in T2DM patients	[177]
Semaglutide	Mansoor Husain	2019	T2DM	Human	Oral semaglutide was non-inferior to vehicle in cardiovascular safety and showed potential benefits in reducing cardiovascular event risk	[178]
Efpeglenatide	Hertzel C Gerstein	2021	T2DM	Human	Efpeglenatide significantly decreased the incidence of major adverse cardiovascular events (MACE) in T2DM patients	[179]
Albiglutide	Adrian F Hernandez	2018	T2DM	Human	Albiglutide markedly reduced the risk of major cardiovascular events in T2DM patients with established cardiovascular disease	[180]
Liraglutide	Jennifer B Green	2024	T2DM	Human	Compared with other commonly used anti-hyperglycemic medications, liraglutide was associated with reduced cardiovascular event risk in relatively lower-risk T2DM patients	[181]

**Table 2:** Preclinical studies of GLP1RAs for the treatment of DM or DM-related diseases

Generic name	First author	Year	Disease	Model	Findings	Refs.
Liraglutide	Xiaoqing Yan	2022	DM with hindlimb ischemia (HLI)	Mice	Under diabetic conditions, liraglutide effectively promotes ischemic angiogenesis and improves endothelial progenitor cells (EPCs) function	[182]
	Tzu-Hsien Tsai	2019	DM with vascular injury	Mice	Liraglutide attenuates neointima formation in diabetic mice by inhibiting endothelial-to-mesenchymal transition (EndMT)	[183]
	Hideki Kushima	2017	DM with arterial restenosis	Mice	Liraglutide stimulates endothelial NO production in ex vivo hyperglycemic environments and exerts anti-restenotic effects in mice with severe hyperglycemia	[184]
Oral nanomicelle formulation of liraglutide	Laxman Subedi	2025	T2DM	Mice	Orally administered liraglutide nanomicellar formulation significantly reduces fasting blood glucose and HbA1c levels in diabetic models by enhancing insulin sensitivity and modulating lipid metabolism	[185]
Semaglutide	Kaibin Lin	2024	DM	Mice	Semaglutide demonstrates cardioprotective effects against diabetic heart failure (HF) by alleviating cardiac inflammation through the Sirt3-dependent RKIP signaling pathway	[186]

#### Sodium-glucose cotransporter 2 inhibitors(SGLT2is)

SGLT2is exert their glucose-lowering effects by inhibiting the sodium-glucose cotransporter 2 (SGLT2) receptor in the renal proximal tubule, which reduce glucose reabsorption and promote urinary glucose excretion [187]. Their antihyperglycemic mechanism is characterized by insulin independence, while osmotic diuresis contributes to reduced blood volume and blood pressure, ultimately improving cardiometabolic risk. Multiple meta-analyses have demonstrated that [188, 189], compared with vehicle treatment, SGLT2i therapy significantly reduces the risk of cardiovascular disease in patients with T2DM. Specifically, empagliflozin was associated with a 38% relative risk reduction in cardiovascular mortality, a 31% reduction in sudden death, and a 35% decrease in hospitalization for HF [190].

SGLT2i intervene in pathological vascular remodeling through multiple pathways. Studies have demonstrated that [191] empagliflozin suppresses the proliferation and migration of VSMCs by downregulating PDGF-related signaling pathways, including inhibition of PDGF receptor β, Akt, and signal transducer and activator of transcription 3 (STAT3) phosphorylation. Furthermore, empagliflozin attenuates interleukin-17 A (IL-17 A)-induced proliferation and migration of human aortic smooth muscle cells by targeting the TNF receptor-associated factor 3-interacting protein 2 (TRAF3IP2)/ROS/NLRP3/caspase-1-dependent secretion of IL-1β and interleukin-18 (IL-18), thereby restoring vascular homeostasis [192]. The regulatory role of SGLT2i in the phenotypic transition of VSMCs has gradually gained indirect supporting evidence through clinical studies. Cardiovascular outcome trials, including EMPA-REG OUTCOME, DECLARE-TIMI 58, have demonstrated that empagliflozin and dapagliflozin significantly reduce the risk of MACE and HF hospitalization in patients with T2DM [190, 193]. This effect is partially attributed to the inhibition of vascular remodeling. Imaging studies further demonstrated that SGLT2i treatment improved arterial stiffness and carotid intima-media thickness (CIMT), effects which were closely associated with the restoration of VSMCs contractile function and suppression of inflammation [194–197]. Although clinical data directly addressing phenotypic transition in VSMCs remain to be further investigated, current evidence suggests that SGLT2i improves vascular biological function through pleiotropic mechanisms, providing a novel dimension for the management of diabetic macrovascular complications (Tables 3 and 4).

**Table 3:** Clinical studies of SGLT2is for the treatment of DM or DM-related diseases

Generic name	First author	Year	Disease	Model	Findings	Refs.
Dapagliflozin	Mats Christian Højbjerg Lassen	2024	T2DM and HF	Human	In patients with T2DM and HF with left ventricular ejection fraction (LVEF) > 40%, dapagliflozin demonstrated a safe and sustained reduction in the risk of HF worsening and cardiovascular death, irrespective of background glucose-lowering therapy	[198]
	Dennis D Wang	2024	T2DM	Human	Dapagliflozin treatment for 12 months reduced systemic inflammation in patients with T2DM	[199]
	Andrei C Sposito	2021	T2DM	Human	Regardless of glycemic control, dapagliflozin improved microvascular and macrovascular endothelial function in patients with T2DM and subclinical carotid atherosclerotic disease	[194]
Empagliflozin	Roy Hershenson	2024	T2DM with CAD	Human	Empagliflozin conferred cardiovascular protection by enhancing the levels and functionality of circulating endothelial progenitor cells in patients with T2DM and stable CAD	[200]
	David Fitchett	2024	T2DM and cardiovascular disease [mostly atherosclerotic (ASCVD)]	Human	In patients with T2DM and ASCVD, empagliflozin reduced the risk of MI	[201]
	Phyo T Htoo	2024	T2DM	Human	Compared with DPP-4i, empagliflozin significantly decreased the incidence of MI or stroke in T2DM patients, with a greater absolute risk reduction observed in elderly individuals and those with a history of ASCVD or HF	[202]
	Adrian F Hernandez	2024	T2DM and acute myocardial infarction	Human	Empagliflozin was associated with a reduced risk of HF in patients with left ventricular dysfunction or congestion following acute myocardial infarction	[203]
Canagliflozin	Abhinav Sharma	2024	T2DM with and without prevalent cardiovascular disease	Human	Canagliflozin lowered the risk of MACE, including myocardial infarction, stroke, and HF, in T2DM patients at high cardiovascular risk	[204]
	Seshagiri Rao Nandula	2021	T2DM	Human	Canagliflozin reduced cardiovascular disease risk by enhancing EPCs migration in T2DM patients	[205]

**Table 4:** Preclinical studies of SGLT2is for the treatment of DM or DM-related diseases

Generic name	First author	Year	Disease	Model	Findings	Refs.
Dapagliflozin	Sven O Göpel	2024	T2DM	Mice and monkeys	Dapagliflozin improves vascular function via a glucagon signaling-dependent pathway	[206]
	Yafei Jiang	2024	T2DM	Mice	Dapagliflozin-remodeled gut microbiota promotes β-cell regeneration in T2DM mice by enhancing GLP-1 secretion	[207]
	Qingqing Wu	2023	Heart failure with preserved ejection fraction(HFpEF)	Mice	Dapagliflozin ameliorates MI and transverse aortic constriction (TAC)-induced HF independently of SGLT2	[208]
	Ying Zhou	2023	T2DM	Mice	Dapagliflozin confers macrovascular benefits by attenuating oxidative stress to prevent endothelial senescence and dysfunction	[209]
	Weiyue Zhang	2023	DM with wounds	Mice	Dapagliflozin-loaded exosome-mimetic vesicles promote angiogenesis in diabetic mice by targeting ECs and enhancing their functionality	[210]
Empagliflozin	Jing-xuan Han	2023	DM with HLI	Mice	Empagliflozin suppresses hyperglycemia-induced ferroptosis in a GPX4-dependent manner and facilitates revascularization in diabetic HLI model mice	[211]
	Juliana Mira Hernandez	2024	T2DM with HFpEF	Mice	Empagliflozin attenuates arrhythmias in HFpEF by inhibiting Ca^2+^/calmodulin-dependent protein kinase II (CaMKII) signaling	[212]
	Xiao-Xue Li	2024	T2DM	Mice	Empagliflozin ameliorates vascular calcification by suppressing aberrant activation of NLRP3 inflammasome in the aortic smooth muscle layer of T2DM mice	[213]
	Ellen Vercalsteren	2024	T2DM with transient middle cerebral artery occlusion (tMCAO)	Mice	Post-stroke intervention with empagliflozin normalizes parenchymal pericyte density in the infarct core of T2DM mice	[214]

#### Metformin

Metformin, a first-line anti-hyperglycemic medication for T2DM, primarily exerts its therapeutic effects by inhibiting hepatic gluconeogenesis, enhancing peripheral tissue insulin sensitivity, and modulating intestinal glucose metabolism [215, 216]. The UKPDS 34 demonstrated that metformin treatment significantly reduced the risk of MI in obese diabetic patients (hazard ratio reduction: 39%), with its cardiovascular benefits partially attributed to improved vascular function [217]. Cohort studies further revealed that, compared to sulfonylurea monotherapy, metformin monotherapy was associated with a one-third lower risk of mortality due to major cardiovascular events [218], accompanied by significant reductions in vascular inflammatory markers [high-sensitivity C-reactive protein (hs-CRP) and IL-6] [219]. Collectively, these findings suggest that metformin holds potential for the management of diabetic macrovascular complications.

Studies have shown that metformin inhibits high glucose-induced proliferation and migration of VSMCs through AMPK-dependent pathway. The underlying mechanism involves inducing phosphorylation of PDZ and LIM domain 5 (Pdlim5) and activating the AMPK-Pdlim5 pathway to disrupt VSMCs migration [220]. Furthermore, as both a methylglyoxal scavenger and AGEs inhibitor [221], metformin reduces ROS through peroxisome proliferator-activated receptor γ coactivator-1-alpha (PGC-1α) to prevent osteogenic phenotypic transition in VSMCs [222]. Collectively, these findings support metformin’s multifaceted protective effects against diabetic vascular pathology (Tables 5 and 6).

**Table 5:** Clinical studies of Metformin for the treatment of DM or DM-related diseases

Generic Name	First Author	Year	Disease	Model	Findings	Refs.
Metformin	Johanna M G Stultiens	2022	T2DM	Human	No clinically relevant evidence was found to indicate that metformin treatment exerts significant effects on cardiac biomarker levels [including cardiac troponin I (cTnI) and cardiac troponin T (cTnT)] when compared with vehicle control	[223]
	Celestino Sardu	2019	prediabetes	Human	Metformin may reduce cardiovascular event risk in prediabetic patients by ameliorating coronary endothelial dysfunction	[224]
	Aurélien Mary	2017	T2DM	Human	In patients with T2DM and high cardiovascular risk, metformin treatment demonstrated an independent inverse correlation with the severity of lower extremity arterial calcification	[225]
	Anders Hostrup Larsen	2020	HFpEF	Human	For non-diabetic patients with HFrEF, metformin improves myocardial efficiency by reducing myocardial oxygen consumption	[226]
	Wiebe M C Top	2022	T2DM	Human	The potential beneficial effects of metformin on endothelial function cannot be explained solely by reductions in asymmetric dimethylarginine (ADMA) or improvements in global arginine availability. Its putative effects on the nitric oxide pathway extend beyond the specific metabolites investigated in this study	[227]

**Table 6:** Preclinical studies of Metformin for the treatment of DM or DM-related diseases

Generic Name		First Author	Year	Disease	Model	Findings	Refs.
Metformin	Sathish Babu Vasamsetti		2015	AS	Mice	Metformin attenuates STAT3 phosphorylation via AMPK activation to suppress inflammation and monocyte-to-macrophage differentiation	[228]
	Sabrina Robichaud		2022	AS	Mice	Metformin activates autophagy in VSMC-derived foam cells to facilitate cholesterol efflux to high-density lipoprotein (HDL)	[229]
	Chak Kwong Cheng		2023	AS	Mice	SOX4 as a novel phenotypic regulator exacerbates atherosclerotic formation during endothelial dysfunction, whereas metformin suppresses SOX4 levels in ECs	[230]
	Caleb A Padgett		2023	Obesity	Mice	Metformin ameliorates vascular dysfunction by reducing endothelial galectin-3 (GAL3) expression via improved glycated hemoglobin (HbA1c) levels in obese mice	[231]
	Fan Yang		2019	diabetic cardiomyopathy (DCM)	Mice	Metformin protects cardiac function by inhibiting the NLRP3 inflammasome through AMPK-mediated autophagy activation	[232]
	Khondker Ayesha Akter		2024	Ischemic stroke	Mice	Metformin ameliorates neuroinflammation in ischemic stroke by activating the Nrf2-ARE pathway to suppress NF-κB signaling	[233]
	Jin Young Sung		2024	AS	Mice	Metformin attenuates atherosclerotic and senescent phenotypes in VSMCs through AMPK-dependent enhancement of PGC-1α phosphorylation	[234]
	Xiaoyun Zhao		2023	Obesity	Mice	Enhancement of branched-chain amino acid (BCAA) catabolism or reduction of BCAA intake potentiates the glucose-lowering efficacy of metformin	[235]
	Yujiao Zhang		2023	AS	Mice	Metformin alleviates intracellular lipid overload, restores macrophage viability and autophagic flux ex vivo, and ultimately abolishes the pro-atherogenic effects of Card9 deficiency in vivo	[236]
	Gang Wang		2022	T2DM	Mice	Metformin prevents methylglyoxal (MGO)-induced apoptosis by suppressing oxidative stress both ex vivo and in vivo	[237]

#### Thiazolidinediones (TZDs)

TZDs, including rosiglitazone and pioglitazone, lower blood glucose levels primarily by activating peroxisome proliferator-activated receptor gamma (PPAR-γ), which enhances insulin sensitivity and regulates lipid metabolism. Their hypoglycemic effects arise from three key mechanisms: promoting adipocyte differentiation, increasing adiponectin secretion, and inhibiting hepatic gluconeogenesis [238].

Interestingly, PPAR-γ agonists exhibit bidirectional regulation on the phenotypic transition of VSMCs. Fundamental studies have demonstrated that PPAR-γ agonists maintain the contractile phenotype of VSMCs by downregulating profibrotic genes [e.g., connective tissue growth factor(CTGF)] through inhibition of the TGF-β/Smad3 signaling pathway [239]. Concurrently, they suppress VSMCs growth and proliferation by elevating the levels of cyclin-dependent kinase inhibitor p27 [240]. However, clinical studies have yielded conflicting results: The PROactive trial demonstrated that pioglitazone reduced the risk of cardiovascular events in patients with T2DM (16% reduction in secondary endpoint risk), but its effect on VSMCs calcification remains controversial [241]. Conversely, the RECORD study suggested that rosiglitazone might increase the risk of HF, though the underlying mechanisms remain unclear [242]. In summary, PPAR-γ agonists exhibit a dual “metabolic benefit-local risk” characteristic in modulating VSMC phenotype, and their precise clinical application requires careful consideration of target organ effects and molecular dose-dependency (Tables 7 and 8).

**Table 7:** Clinical studies of TZDs for the treatment of DM or DM-related diseases

Generic Name	First Author	Year	Disease	Model	Findings	Ref.
Saroglitazar	Manjunath Krishnappa	2020	T2DM	Human	Saroglitazar effectively improved glycemic control and lipid parameters over 56 weeks and demonstrated promising potential in reducing cardiovascular risk in patients with T2DM	[243]
Pioglitazone	Ursula White	2021	Obesity	Human	Pioglitazone may exert beneficial metabolic effects by promoting the formation of new adipocytes in subcutaneous adipose tissue, thereby inducing differential adipogenesis in obese women	[244]
	Yury O Nunez Lopez	2022	T2DM	Human	Pioglitazone reduces circulating EVs miR-195-5p levels while increasing its expression in adipose tissue, leading to improved inflammatory status, glycemic control, and insulin sensitivity in T2DM patients	[245]
Rosiglitazone	Marjorie Bastien	2019	T2DM	Human	Although rosiglitazone improves glycemic control and metabolic disorders in patients with T2DM and CAD, it may lead to reduced aerobic exercise capacity due to increased body weight and subcutaneous fat accumulation	[246]
	H Florez	2015	T2DM	Human	In elderly patients with T2DM, rosiglitazone use is associated with a reduced risk of MACE and cardiovascular mortality, without increasing the incidence of myocardial infarction	[247]

**Table 8:** Preclinical studies of TZDs for the treatment of DM or DM-related diseases

Generic Name	First Author	Year	Disease	Model	Findings	Ref.
Rosiglitazone	Theresa V Rohm	2024	Obesity	Mice	Rosiglitazone induces polarization of adipose tissue macrophages toward the M2 phenotype and promotes the secretion of miRNA-containing EVs. These EVs circulate to insulin-target tissues, thereby enhancing insulin sensitivity, glucose homeostasis, and β-cell insulin secretion, a process dependent on transporters such as miR-690	[248]
	Mirian Krystel De Siqueira	2024	Obesity	Mice	By selectively promoting the translation of mRNAs encoding pathways related to adipogenesis and lipid metabolism, rosiglitazone not only drives the transcriptional network of adipocytes but also ensures their functional specialization	[249]
	Young Jae Bahn	2024	Obesity	Mice	Rosiglitazone and TGF-β receptor antagonists exhibit synergistic effects in suppressing adipose tissue fibrosis and promoting beige adipogenesis	[250]
	Lingling Yu	2023	T2DM	Mice	Rosiglitazone mitigates ECs activation and injury by reducing monocyte adhesion and cytokine secretion while simultaneously upregulating heat shock protein 22 (HSP22) expression	[251]
Pioglitazone	Jie Wen	2023	Obesity	Mice	Pioglitazone elevates miR-182-5p levels in the subcutaneous white adipose tissue of mice	[252]

### Pharmacological targeting of VSMCs

In the pathological progression of diabetic macrovascular disease, aberrant phenotypic plasticity of VSMCs serves as a central mechanism driving AS, vascular calcification, and restenosis. Although conventional therapeutic strategies (e.g., lipid-lowering and anti-inflammatory therapies) can delay plaque progression, they fail to reverse the pathological phenotypic transformation of VSMCs (e.g., synthetic, osteogenic, and foam-cell phenotypes), leading to uncontrolled vascular remodeling. Recent pharmacological interventions targeting VSMC-specific molecular pathways have rapidly emerged, focusing on three key regulatory dimensions: epigenetic reprogramming, metabolic remodeling, and mechanosensing. These strategies fundamentally modulate VSMCs fate determination, providing a precision medicine paradigm to disrupt the vicious cycle of “vulnerable plaque–vascular calcification–post-interventional restenosis”.

#### MicroRNA(miRNA) intervention strategy

Under high-glucose conditions, the dynamic imbalance of non-coding RNAs (ncRNAs) drives AS and vascular calcification by regulating the phenotypic plasticity of VSMCs. To address this imbalance, pharmacological interventions focus on restoring the homeostasis of the ncRNA network. For instance, intravenous delivery of lentiviral miR-145 reduces Krüppel-like factor 4 (KLF4) expression while increasing myocardin expression in the aortas of ApoE^(−/−)^ mice, thereby reversing VSMCs dedifferentiation and attenuating plaque burden [253]. Both miR-181a-5p and miR-181a-3p mimics were demonstrated to attenuate AS progression by suppressing NF-κB activation and vascular inflammation through targeting Table 2 and NEMO, respectively [254]. A correlation was observed between miR-34a and Runx2 expression in both young and aged mice. Mechanistically, miR-34a was found to induce VSMCs senescence by downregulating SIRT1. Notably, miR-34a deficiency significantly reduced soft tissue and aortic medial calcification [255]. These findings suggest that pharmacological inhibition of miR-34a may represent a potential therapeutic strategy to restore SIRT1 activity and consequently delay vascular calcification. Additionally, the antisense non-coding RNA in the INK4 locus (ANRIL) can regulate the proliferation and apoptosis of VSMCs through distinct transcriptional splicing mechanisms. For instance, linear ANRIL modulates VSMCs proliferation within plaques via chromatin remodeling and transcriptionally influences macrophage proliferation and apoptosis. In contrast, circular ANRIL affects VSMC proliferation and apoptosis through both chromatin modification and interference with ribosomal RNA (rRNA) maturation [256].

It is noteworthy that although lipid nanoparticles (LNPs) and adeno-associated virus (AAV) delivery systems have demonstrated potential in preclinical studies, their limited tissue targeting specificity and potential immunogenicity remain critical translational bottlenecks. Future strategies may involve combining exosome engineering modifications (e.g., CD47 labeling to enhance vascular targeting) with epigenetic editing technologies (e.g., dCas9-mediated promoter methylation regulation) to achieve spatiotemporal-specific modulation of non-coding RNA networks, thereby providing multidimensional therapeutic approaches for diabetic macrovascular complications.

#### Metabolic reprogramming

Metabolic reprogramming of VSMCs plays a pivotal role in mitigating diabetic macrovascular complications, involving multiple critical molecular targets and signaling pathways, particularly their alterations in energy metabolism, inflammatory responses, and cell fate determination.

First, the remodeling of glucose metabolism is essential for VSMCs function. The expression regulation of hexokinase and pyruvate dehydrogenase kinase (PDK) directly influences cellular glucose metabolism and energy production. Studies have demonstrated that interventions targeting these key enzymes can improve insulin sensitivity in VSMCs, thereby reducing cellular stress induced by hyperglycemia and mitigating the risk of macrovascular complications [257–259]. Furthermore, the regulation of lipid metabolism constitutes a critical component of VSMCs metabolic remodeling. Activation of fatty acid synthase (FASN) and PPAR-γ not only modulates lipid storage but also confers vascular endothelial protection through attenuation of inflammatory responses [260–262]. In diabetic patients, this regulatory role may potentially alleviate both microvascular and macrovascular complications arising from endothelial dysfunction. In addition, mitochondrial function plays a pivotal role in the metabolic remodeling of VSMCs. The maintenance of mitochondrial dynamics, energy production, and redox homeostasis is critical for preventing the pathological transformation of VSMCs. By modulating mitochondrial biogenesis and autophagy pathways, such as through AMPK activation, VSMCs can maintain a healthier metabolic state, thereby reducing apoptosis and necrosis and subsequently attenuating atherosclerotic plaque formation [263–266]. Furthermore, the regulation of inflammatory and signaling pathways, including NF-κB and TGF-β, has been demonstrated to significantly influence VSMCs’ metabolic remodeling [267]. Optimizing the modulation of these signaling pathways can effectively mitigate inflammatory responses, thereby reducing vascular damage and subsequent complications induced by chronic inflammation.

From a comprehensive perspective, the metabolic remodeling of VSMCs not only involves the regulation of glucose and lipid metabolism, but also closely associated with the mitigation of inflammation, improvement of mitochondrial function, and maintenance of normal cellular viability. These mechanisms collectively promote vascular health and exert substantial protective effects in alleviating macrovascular complications induced by DM.

#### Regulation of mechanical stress signaling

In diabetic macrovascular complications, pharmacological strategies targeting the mechanosignaling network of VSMCs are emerging as a novel therapeutic focus for vascular remodeling intervention. Aberrant mechanical stresses (e.g., disturbed local shear stress at plaque sites and elevated circumferential tension) drive pathological phenotypic switching of VSMCs by mediating Rac-Rho homeostasis [268] and Yes-associated protein (YAP) activation [269]. The hyperglycemic milieu further amplifies the pro-fibrotic effects of these mechanical signals.

**Fig. 5:**
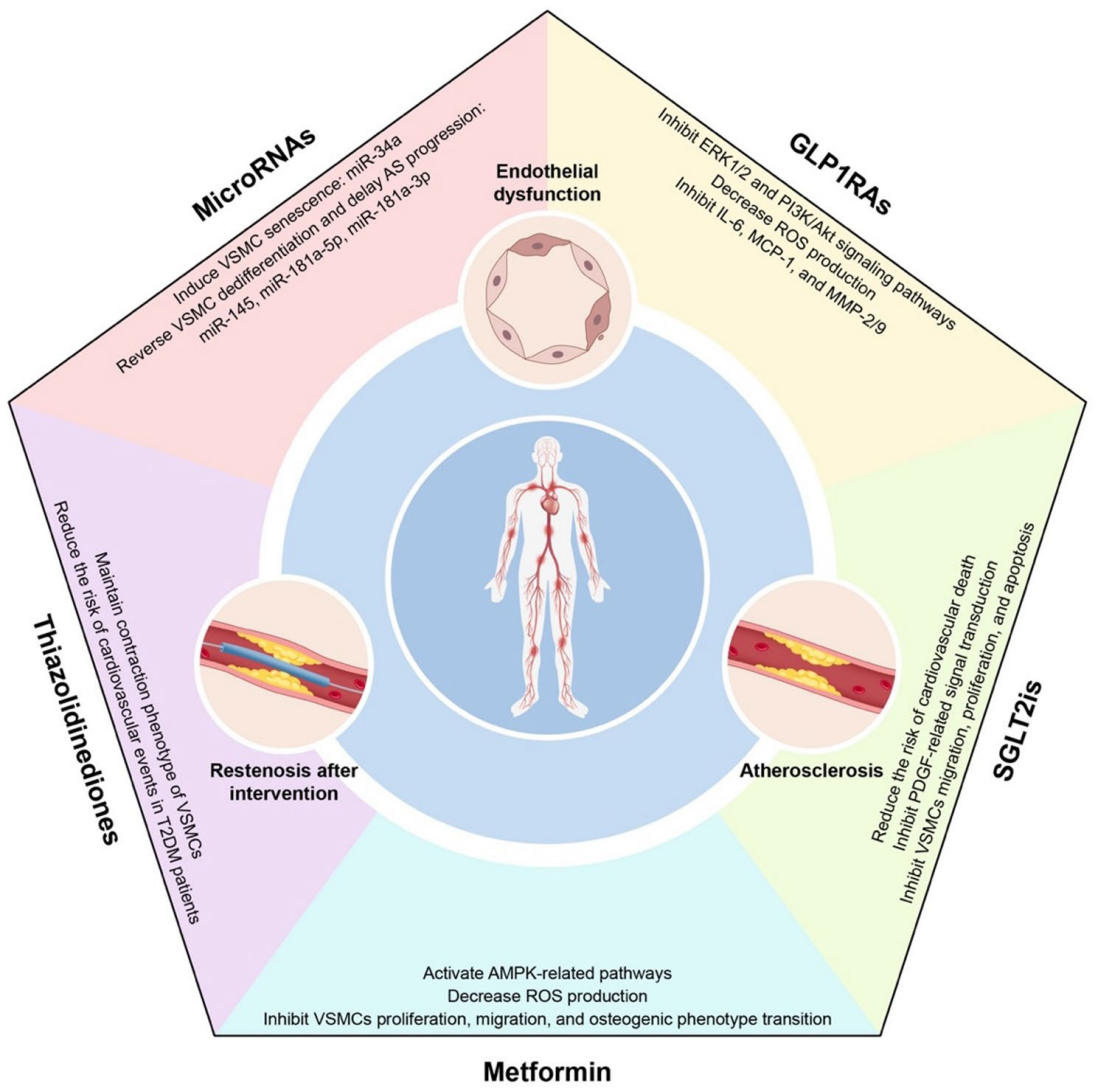
**Schematic Overview** Endothelial dysfunction, AS, and post-interventional restenosis represent three core mechanisms underlying macrovascular complications in diabetes mellitus, with VSMCs potentially playing a pivotal role in these processes. Four classes of pharmacological agents—GLP-1RAs, SGLT2is, metformin, and thiazolidinediones—may exert therapeutic effects by modulating the phenotypic transition of VSMCs. Additionally, miRNAs can regulate VSMCs behavior, representing a promising research direction for the treatment of diabetic macrovascular complications. Abbreviations: ERK1/2: extracellular regulated protein kinases 1/2, PI3K/Akt: phosphatidylinositol 3-kinase/protein kinase B, ROS: reactive oxygen species, IL-6: interleukin-6, MCP-1: monocyte chemoattractant protein-1, MMP-2/9: matrix metalloproteinase-2/9, PDGF: platelet-derived growth factor, VSMCs: vascular smooth muscle cells, AMPK: AMP-activated protein kinase, T2DM: type 2 diabetes mellitus, AS: atherosclerosis

Notably, the small-molecule agonist Yoda1, which targets the mechanosensitive ion channel Piezo1, has been shown to modulate the lipid microenvironment surrounding channel proteins [270, 271] and attenuate calcium influx, thereby significantly delaying the progression of arterial calcification [272]. Furthermore, nanotechnology-based local delivery systems [273](e.g., YAP siRNA-loaded liposomes) can specifically suppress the aberrant mechanical responses of VSMCs within plaques by enhancing vascular wall targeting, although the precise mechanisms and clinical translation require further investigation. Despite challenges such as tissue selectivity and adaptation to dynamic mechanical microenvironments, these strategies offer innovative approaches to reverse the pathological progression of diabetic macrovascular complications from a mechanobiological perspective by reconstructing the “mechanical signaling-metabolic reprogramming” interaction network (Fig. 5).

## Conclusion

The pathogenesis of diabetic macrovascular complications is a complex process involving pathological remodeling of VSMCs under the dual influence of metabolic disturbances and mechanical stress. In this review, we focus on the interplay among vascular endothelial dysfunction, inflammatory responses, and calcification processes. We highlight that hyperglycemia drives the transformation of VSMCs into a pro-inflammatory and pro-fibrotic phenotype by inducing metabolic reprogramming and aberrant activation of mechanosensitive signaling pathways. This phenotypic shift further promotes the formation and instability of AS plaques, providing a systematic perspective for understanding the molecular mechanisms underlying diabetic macrovascular complications. In recent years, targeted intervention strategies such as YAP/TAZ inhibitors (e.g., verteporfin) and siRNA nanodelivery systems have demonstrated promising therapeutic efficacy by specifically modulating key molecular nodes. However, their clinical applications remain limited by insufficient targeting precision and low delivery efficiency. Future research should focus on the following breakthroughs: (1) elucidating the mechano-metabolic coupling mechanisms through multi-omics technologies and biomimetic models; (2) developing more precise targeted delivery systems; and (3) establishing personalized treatment regimens based on patient heterogeneity. These advancements will provide novel theoretical foundations and therapeutic strategies for the precision management of diabetic macrovascular complications, thereby accelerating the translation of basic research into clinical applications in this field.

## Data Availability

Not applicable.

## Abbreviations

AAV: Adeno-associated virus
ADMA: Asymmetric dimethylarginine
AGEs: Advanced glycation end products
AMPK: AMP-activated protein kinase
Ang II: Angiotensin II
ANRIL: Antisense non-coding RNA in the INK4 locus
AS: Atherosclerosis
ASCVD: Cardiovascular disease mostly atherosclerotic
α-SMA: α-Smooth muscle actin
BCAA: Branched-chain amino acid
CAD: Coronary artery disease
CaMKII: Ca^2+^/calmodulin-dependent protein kinase II
CIMT: Carotid intima-media thickness
CTGF: Connective tissue growth factor
cTnI: Cardiac troponin I
cTnT: Cardiac troponin T
CVD: Cardiovascular disease
DCBs: Drug-coated balloons
DCM: Diabetic cardiomyopathy
DESs: Drug-eluting stents
DM: Diabetes mellitus
ECM: Extracellular matrix
ECs: Endothelial cells
ELK1: ETS domain-containing protein-1
EndMT: Endothelial-to-mesenchymal transition
eNOS: Endothelial nitric oxide synthase
EPCs: Endothelial progenitor cells
ER: Endoplasmic reticulum
ET-1: Endothelin-1
EVs: Extracellular vesicles
FASN: Fatty acid synthase
GAL3: Galectin-3
GLP-1: Glucagon-like peptide 1
GLP1RA: Glucagon-like peptide 1 receptor agonist
GPx: Glutathione peroxidase
Grb10: Growth factor receptor-bound protein 10
HbA1c: Glycated hemoglobin
HDL: High-density lipoprotein
HF: Heart failure
HFpEF: Heart failure with preserved ejection fraction
HLI: Hindlimb ischemia
HMGB2: High-mobility group box 2
hs-CRP: High-sensitivity C-reactive protein
HSP22: Heat shock protein 22
IFN-γ: Interferon-gamma
IL-1β: Interleukin-1 beta
IL-6: Interleukin-6
IL-17A: Interleukin-17A
IL-18: Interleukin-18
ISR: In-stent restenosis
KLF2: Krüppel-like factor 2
KLF4: Krüppel-like factor 4
LDL: Low-density lipoprotein
LNP: Lipid nanoparticle
LVEF: Left ventricular ejection fraction
MACE: Major adverse cardiovascular event
MAPK: Mitogen-activated protein kinases
MCP-1: Monocyte chemoattractant protein-1
MGO: Methylglyoxal
MHC: Major histocompatibility complex
MI: Myocardial infarction
miRNA: MicroRNA
MLCK: Myosin light chain kinase
MMP-2: Matrix metalloproteinase-2
MMP-9: Matrix metalloproteinase-9
MMPs: Matrix metalloproteinases
mtDNA: Mitochondrial DNA
mtROS: Mitochondrial reactive oxygen species
ncRNA: Non-coding RNA
NF-κB: Nuclear factor-kappa B
NLRP3: NOD-like receptor protein 3
NO: Nitric oxide
Nrf2: Nuclear factor erythroid 2-related factor 2
ox-LDL: Oxidized low-density lipoprotein
P4HA1: Prolyl 4-hydroxylase subunit alpha-1
PCI: Percutaneous coronary intervention
PDGF: Platelet-derived growth factor
PDK: Pyruvate dehydrogenase kinase
Pdlim5: PDZ and LIM domain 5
PGC-1α: Peroxisome proliferator-activated receptor γ coactivator-1-alpha
PI3Kγ: Phosphatidylinositol 3-kinase gamma
PKC: Protein kinase C
PPAR-γ: Peroxisome proliferator-activated receptor gamma
PXDN: Peroxidasin
RAGE: Receptor of advanced glycation end products
ROS: Reactive oxygen species
rRNA: Ribosomal RNA
Runx2: Runt-related transcription factor 2
SGLT2: Sodium-glucose cotransporter 2
SGLT2i: Sodium-glucose cotransporter 2 inhibitor
SM-MHC: Smooth muscle myosin heavy chain
smRNA-seq: Small RNA sequencing
SOD: Superoxide dismutase
STAT3: Activator of transcription 3
T2DM: Type 2 diabetes mellitus
TAC: Transverse aortic constriction
TGF-β: Transforming growth factor-beta
TLR9: Toll-like receptor 9
TLRs: Toll-like receptors
tMCAO: Transient middle cerebral artery occlusion
TNF-α: Tumor necrosis factor-alpha
TRAF3IP2: TNF receptor-associated factor 3-interacting protein 2
TZD: Thiazolidinedione
UKPDS: UK Prospective Diabetes Study
VEGF: Vascular endothelial growth factor
VSMCs: Vascular smooth muscle cells
YAP: Yes-associated protein
